# Heterologous protein production using euchromatin-containing expression vectors in mammalian cells

**DOI:** 10.1093/nar/gkv475

**Published:** 2015-05-14

**Authors:** Katalin Zboray, Wolfgang Sommeregger, Edith Bogner, Andreas Gili, Thomas Sterovsky, Katharina Fauland, Beatrice Grabner, Patricia Stiedl, Herwig P. Moll, Anton Bauer, Renate Kunert, Emilio Casanova

**Affiliations:** 1 Ludwig Boltzmann Institute for Cancer Research (LBI-CR), Vienna, 1090, Austria; 2 Vienna Institute of BioTechnology, Department of Biotechnology, University of Natural Resources and Life Sciences, Vienna, 1190, Austria; 3 Polymun Scientific GmbH, Klosterneuburg, 3400, Austria; 4 Institute of Pharmacology, Center of Physiology and Pharmacology, Comprehensive Cancer Center, Medical University of Vienna, Vienna, 1090, Austria; 5 The Antibody Lab, Vienna, 1210, Austria

## Abstract

Upon stable cell line generation, chromosomal integration site of the vector DNA has a major impact on transgene expression. Here we apply an active gene environment, rather than specified genetic elements, in expression vectors used for random integration. We generated a set of Bacterial Artificial Chromosome (BAC) vectors with different open chromatin regions, promoters and gene regulatory elements and tested their impact on recombinant protein expression in CHO cells. We identified the *Rosa26* BAC as the most efficient vector backbone showing a nine-fold increase in both polyclonal and clonal production of the human IgG-Fc. Clonal protein production was directly proportional to integrated vector copy numbers and remained stable during 10 weeks without selection pressure. Finally, we demonstrated the advantages of BAC-based vectors by producing two additional proteins, HIV-1 glycoprotein CN54gp140 and HIV-1 neutralizing PG9 antibody, in bioreactors and shake flasks reaching a production yield of 1 g/l.

## INTRODUCTION

Recombinant protein production in mammalian cells is the predominant way of nowadays biologic drug production (1,2). Due to the ever increasing demand for protein therapeutics, new methods decreasing the time and costs in the generation of high-yield producer cell lines are extremely demanded. Regarding time considerations, large scale transient gene expression (TGE) (3–5) is the most rapid way to produce multiple recombinant proteins in tens to hundreds milligram quantities, typically from 1 to 10 l culture volumes. TGE is an attractive method to use mainly for preclinical studies and basic research. Nevertheless, for large scale production of therapeutic proteins stable cell line generation is still the method of choice. Overall protein yield of a producer cell line is highly affected by its health status, longevity and metabolism (6). The recent sequencing of the CHO-K1 genome (7) combined with ‘Omics’ based systematic approaches (i.e. transcriptomics, proteomics and metabolomics) will result in a better understanding of these processes (Reviewed in (8–10)). Results of these studies will help to engineer CHO cells with improved culture characteristics, increased lifespan and production (11). Another aspect strongly impacting protein yield and stability is the nature of the expression vector used to generate producer cell lines (11). During the generation of stable cell lines, plasmid DNA integrates in a random manner into the host cell's genome. Since the site of integration has a major impact on transcriptional activity of the incoming construct (so-called ‘chromatin positional effects’ (12)), expression levels can be low and unpredictable resulting in a high variability between individual clones. Consequently, there is a need to screen very high numbers of clones to identify efficient and stable producers (13). Moreover, high-yield producer cell line generation via transgene amplification using selection systems such as the DHFR or GS system (14) is time-consuming, induces genomic instability and may lead to silencing of transgene expression (15). To circumvent chromatin positional effects, expression vectors can be flanked by cis-regulatory elements which reduce the positional effects and allow stable expression of the transgene. Indeed, ubiquitous chromatin opening elements (UCOEs) (16–18), scaffold/matrix attachment regions (S/MARs) (19,20) and antirepressor (21) elements have been reported to have a beneficial effect on protein expression levels and stability. Alternatively, it is possible to insert the gene of interest (GOI) into a pre-defined locus in the host cell by using recombinase mediated cassette exchange (RMCE) methods (22). A well-chosen locus or so-called ‘hot-spot’ containing euchromatin can insure long-term stable protein production levels. However, only a single copy is integrated using this method which may limit the maximum achievable expression levels, although high antibody yields (up to 1 g/l) have been reported using the RMCE technology (23). In contrast to the previously described strategies, we aimed to create a system where we can combine the advantages of targeted integration in a hot-spot and the flexibility of random integration methods. We reasoned that large expression vectors harboring whole loci containing euchromatin (hot-spots) will not be affected by positional effects and will confer high and stable expression levels. To this end, we explored Bacterial Artificial Chromosomes (BACs) as expression vectors for recombinant protein production in CHO cells. BACs have a large cloning capacity (200–300 kilobase (kb)) and therefore they can accommodate an entire locus with most if not all of the elements that control the expression of a gene. Indeed, BACs have been widely used in the mouse transgenic field because they ensure positional effect independent and copy number dependent expression of a transgene (24–26). According to this, BAC vectors should be ideal tools applied to heterologous protein production in mammalian cells. BAC-based expression vectors containing carefully chosen loci should combine the beneficial effects of a stable genetic environment with the possibility to integrate several vector copies in the cell host, thus boosting the transgene expression and making transgene amplification unnecessary.

In this study we aimed to optimize the use of BAC-based vectors for recombinant protein production in CHO cells. To do this, we first generated a series of BAC-based vectors combining different open chromatin loci, promoters and other gene regulatory elements and tested them with an ‘easy to express’ single polypeptide chain protein: the Fc fragment of human IgG1 (IgG-Fc). Thereafter, we challenged our most efficient constructs and demonstrated the usefulness of BAC-based expression vectors producing two additional complex proteins: CN54gp140, a difficult to produce, heavily glycosylated single polypeptide derived from HIV-1 and PG9, a broadly neutralizing anti-HIV-1 antibody, as a complex protein model assembled from two different polypeptide chains. By using these three different protein models, we could demonstrate that BAC-based expression vectors dramatically reduce the time needed to generate high yield and stable cell lines.

## MATERIALS AND METHODS

### Generation of conventional and BAC-based expression vectors

All plasmids were constructed in pBluescript KS vector (Stratagene) by changing the multi cloning site to a new polylinker region with unique restriction enzyme sites. Restriction enzymes and T4 ligase were purchased from Thermo Fisher Scientific. Cloning was performed in DH10B *E.coli* bacteria. All plasmid sequence information is available upon request.

#### BAC recombineering

**Figure 1.**
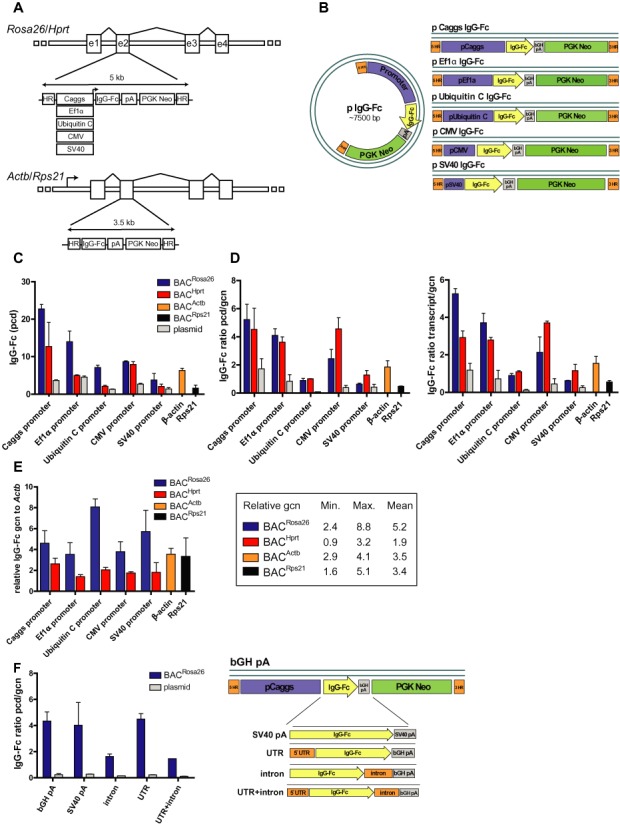
Optimization of BAC-based vector design. IgG-Fc was used as a model protein in every experiment. (**A**) Schematic view of BAC constructs. Incoming constructs were inserted into the second exon (e2) of the *Rosa26*/*Hprt* genes. In BACs containing the *Rosa26* or *Hprt* locus one of the following promoters was used: Caggs, Ef1α, Ubiquitin C, CMV, SV40. pA: polyadenilation signal, PGK Neo: neomycin selection cassette. HR: homology regions for BAC recombineering. In BACs containing the *Actb*/*Rps21* loci the endogenous promoters were used to drive IgG-Fc expression. (**B**) Schematic view of plasmid controls; (**C**) Specific productivity (in pg/cell/day [pcd]) of IgG-Fc producing stable cell pools; (**D**) Specific productivity and relative transcript levels normalized to relative integrated vector (gene) copy numbers (gcn), gcn was calculated relative to genomic *Actb*; (**E**) Integrated gene copy numbers (gcn) relative to *Actb* in the different BAC derived cell pools. Observed minimum (Min.), maximum (Max.) and mean gcn values of the four BAC vectors are shown in a table format. (**F**) Normalized specific productivity of Caggs:BAC^Rosa26^ promoter cell pools containing the bGH polyA, SV40 polyA, an *Rps21*-derived 5′ UTR or an intron (Clontech) sequence in the 3′ position or the combination of both. Schematic view of these constructs is depicted on the right side of the figure, *n* = 2 independent transfections, error bars represent SEM.

BAC recombineering was performed as previously described (27,28). DH10B *E.coli* harboring the corresponding BACs were electroporated with the temperature sensitive pSC101-BAD-gbaA plasmid which carries the recombinase proteins necessary for homologous recombination. Cells harboring the pSC101-BAD-gbaA plasmid were selected in tetracycline (5 μg/ml) at 30°C overnight. Bacterial cells derived from one single positive colony were cultured overnight at 30°C and transferred to 50 ml of fresh medium next day. At an optical density (OD_600_) of 0.2, the expression of the recombinogenic proteins was induced by the addition of L-arabinose (to a 0.3%–0.4% final concentration) and by shifting the temperature to 37°C. After one additional hour, cells were harvested and electro-competent cells were prepared by a double wash with ice cold distilled water. Cells were resuspended in 10% ice cold glycerol solution and aliquoted to Eppendorf tubes (100 μl) prior snap freezing in liquid N_2_ or directly used for electroporation. 50–100 ng of linearized plasmid DNA carrying the insert for BAC modification (Figures 1b and 4a plasmid-controls) flanked by 120–200 base pair (bp) homology regions (HR) to the respective BAC was electroporated to these cells with a Gene Pulser Xcell (BioRad) electroporator. Cells were incubated for 70 min at 37°C without antibiotics in LB medium and plated on LB-agar containing 15 μg/ml kanamycin at 37°C overnight. BAC DNA was isolated from resistant colonies and then analyzed for correct recombination by polymerase chain reaction (PCR) using primer pairs within the incoming construct and the BAC. Original BAC clones were obtained from the BAC PAC Resources Children's Hospital Oakland Research Institute (CHORI). *Rosa26* BAC: RP24–85I15, *Hprt* BAC: RP23–13N1, *Actb* BAC: RP23–249B5, *Rps21* BAC: RP23–16P3.

**Figure 2.**
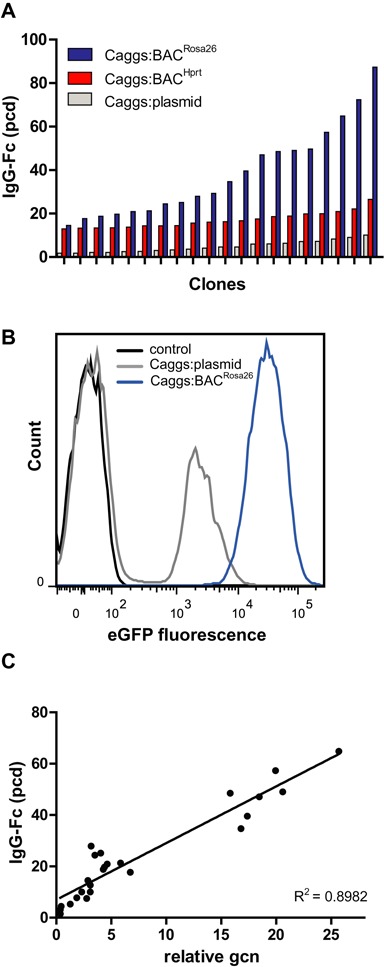
Clonal distribution of IgG-Fc cell pools. (**A**) Specific productivity (pcd) of the 20 best clones out of 50 pre-screened clones derived from stable cell pools generated with the Caggs:BAC^Rosa26^, Caggs:BAC^Hprt^ and Caggs:plasmid vectors. (**B**) FACS analysis shows eGFP fluorescence of Caggs:BAC^Rosa26^ and Caggs:plasmid stable cell pools expressing eGFP compared to empty plasmid control cells. (**C**) Correlation between specific productivity of Caggs:BAC^Rosa26^ clones and their integrated vector (gene) copy numbers (gcn) relative to *Actb* gcn, *n* = 25.

**Figure 3.**
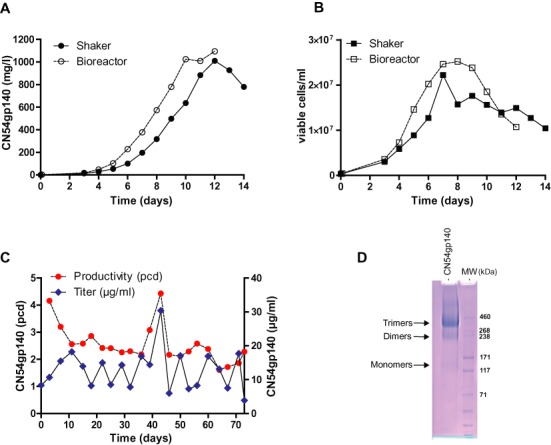
Fed-batch production of CN54gp140 using an optimized BAC-based vector. Fed-batch production of CN54gp140 with the Caggs:BAC^Rosa26^ vector in CHO-K1 cells. (**A**) Product accumulation (mg/l) and (**B**) viable cell densities (viable cells/ml) are compared in shake flasks and in bioreactors. (**C**) Long-term production stability of the Caggs:BAC^Rosa26^ CN54gp140 cell line without selection pressure. (**D**) Quality control of purified CN54gp140 in a non-reducing SDS-PAGE.

**Figure 4.**
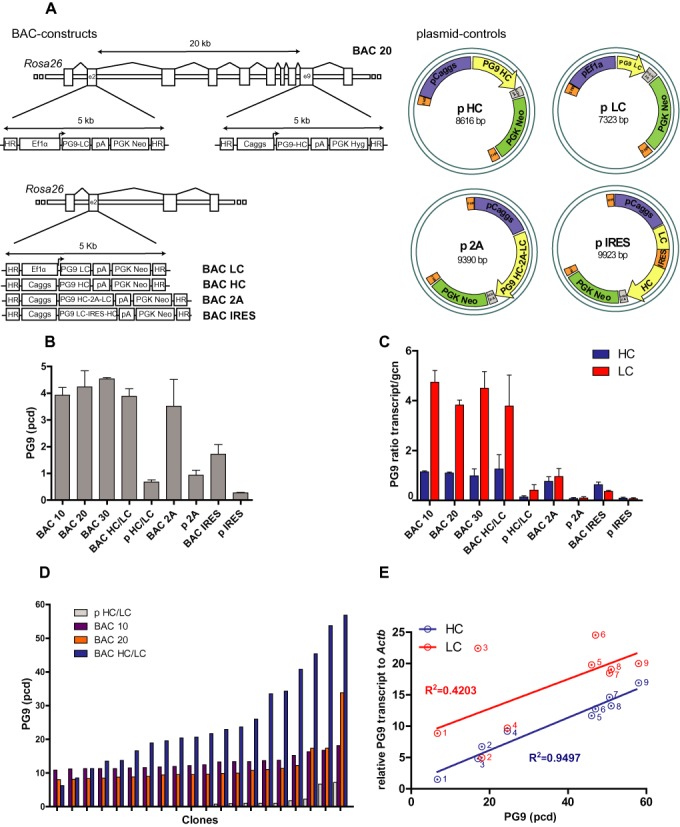
Strategies for production of the anti-HIV-1 antibody PG9 as a complex protein model using BAC-based vectors. (**A**) Schematic view of human IgG1-λ PG9 antibody constructs: BACs and plasmid controls. In the case of BAC 10, BAC 20 and BAC 30 constructs, heavy chain (HC) and light chain (LC) expression units were inserted into the same BAC^Rosa26^ at different locations 10, 20 and 30 kb apart from each other (example shows BAC 20). Lower panel, BAC LC and BAC HC vectors: LC and HC expression cassettes were recombined into two independent BAC^Rosa26^ vectors. Stable cell pools were generated by co-transfection of these two independent BACs (BAC HC/LC). BAC 2A and BAC IRES: LC and HC expression cassette was inserted into a single BAC. LC and HC expression was linked by a bicistronic mRNA using the FMDV 2A peptide or an IRES element. (**B**) Specific productivity of stable PG9 antibody expressing cell pools (pcd) generated with the constructs described above (p HC/LC, p 2A and p IRES are respective plasmid controls). (**C**) Normalized HC and LC transcript levels to the gene copy number relative to genomic *Actb* (gcn) of integrated vectors in the stable cell pools. (**D**) Specific productivity in pcd of the 20 best clones from 50 pre-screened clones derived from the BAC HC/LC, BAC 10, BAC 20 and plasmid HC/LC (p HC/LC) stable cell pools. *n* = 2–4 independent transfections, error bars represent SEM. (**E**) Correlation between HC and LC transcript levels (normalized to *Actb*) and specific productivity (pcd) of nine PG9 BAC HC/LC-derived clones. Specific productivity correlates with HC transcript with an *R*^2^ = 0.9497.

### Generation of CHO-DUKX-B11 cell pools and single cell clones

#### Cell culturing

CHO-DUKX-B11 (ATCC CRL-9096) cells were maintained in ProCHO5 medium (Lonza) supplemented with 4 mM L-glutamine (PAA), Pen/Strep (Life Technologies), HT-supplement (Invitrogen) and phenol red (Sigma-Aldrich). Cells were cultured in a 37°C, 5% CO_2_, humidified incubator without shaking.

#### Transfections

Lipofection: BACs and control plasmids were used for transfection with Freestyle MAX Reagent (Invitrogen). Briefly, 6.4 μg DNA and 16 μl Freestyle MAX Reagent (1:2.5 DNA : Freestyle MAX Reagent ratio) were incubated together in Opti-MEM (Life Technologies) serum free medium for 12 minutes and added to 2×10^6^ CHO-DUKX-B11 cells plated in 2 ml of ProCHO5 medium (Lonza) with supplements (see above) in 6-well tissue culture plates (Greiner Bio-One). BAC DNA was linearized prior to transfection by overnight digestion with the PI-SceI homing endonuclease (New England Biolabs). Antibiotic selection was started 2 days after transfection using 200 μg/ml geneticin disulfate G418 (Carl Roth) and increased to 400 μg/ml 14 days after transfection. Stable cell pools were analyzed 21 days after transfection.

Electroporation: CHO-DUKX-B11 cells were washed once with sterile 1x DPBS (Gibco) and then were resuspended in electroporation buffer (BioRad). 10 μg of plasmid or BAC DNA was electroporated into 4×10^5^ CHO-DUKX-B11 cells in 200 μl total volume with a Gene Pulser Xcell (BioRad) electroporator. For pulsing the following protocol was used with 2 mm gap cuvettes: 160 V, 15 ms, square wave pulse. After electroporation cells were quickly transferred to 2 ml prewarmed growth media and were cultured as described above.

#### Cell counting and viability

CHO-DUKX-B11 cells were treated for 10 min, 37°C with Accumax (Sigma-Aldrich) solution prior to counting in order to dissociate cell clumps. Cells were counted then with CASY Cell Counter (Model TT, Roche).

#### Clone selection and stability

Single cell clones were obtained by limiting dilution technique. Cells from stable cell pools were plated to 96-well tissue culture plates (Thermo Fisher Scientific) in a 0.5 cell per well density. As sub-cloning medium 50% of 0.22 μm sterile filtered culture supernatant derived from CHO-DUKX-B11 cells and 50% fresh ProCHO5 medium with supplements (see above) containing 400 μg/ml G418 was used. Culture supernatants of growing cells were pre-screened by ELISA. Clones from wells showing a high product concentration were subsequently expanded and analyzed for their specific productivities.

Clonal stability of different CHO-DUKX-B11 cell lines was monitored in T-25 flasks (Greiner Bio-One). Cells were cultured as described above without any antibiotic pressure. Cells were passaged into fresh medium every 3–4 days to 1×10^5^ cells/ml. Specific and volumetric productivity was measured once per week.

### Generation of CHO-K1/PG9 and CHO-K1/CN54gp140 cell lines

The two production clones CHO-K1/PG9 and CHO-K1/CN54gp140 were generated from a serum-free adapted host cell line derived from CHO-K1 (ATCC CCL-61). Transfection was performed using Lipofectin (Life Technologies). Briefly, 5 μg of PI-SceI (New England Biolabs) linearized BAC DNA was complexed with 25 μl Lipofectin in CD-DG44 medium (Life Technologies) containing 8 mM L-glutamine and 0.18% Pluronic F68 in a total volume of 200 μl. One million host cells, grown in CD-CHO medium (Life Technologies) supplemented with 8 mM L-glutamine were harvested in exponential growth phase and resuspended in 1.8 ml CD-DG44 medium with supplements. DNA complexes were added drop-wise to the cells and the transfection mixture was incubated in a humidified CO_2_ shaking incubator (ISF1-X; Kuhner) for 24 hours at 37°C, 5% CO_2_ and 125 revolutions per minute (rpm). 18 ml CD-CHO medium supplemented with 8 mM L-glutamine and 500 μg/ml G418 was added to the transfection mix and the culture was seeded in 96-well culture plates (Nunc, Thermo Fisher Scientific) at a volume of 100 μl per well. Plates were incubated in a humidified incubator at 37°C and 5% CO_2_ until growth was observed (14–21 days). Positive wells were analyzed by ELISA. Clones from wells showing a high product concentration were subsequently expanded to a volume of 10 ml in T-25 cell culture flasks (Greiner Bio-One). The clones were monitored in routine culture for 3 passages before the best clone in terms of specific productivity and growth was used for single-cell dilution sub-cloning. Sub-cloning medium was prepared using 50% of 0.22 μm sterile filtered culture supernatant and 50% fresh CD-CHO medium supplemented with 8 mM L-glutamine and 500 μg/ml G418. Cells were diluted in sub-cloning medium and one cell per well was seeded in a 384-well cell culture plate (Corning) in a volume of 50 μl per well. Plates were incubated in a humidified incubator at 37°C and 5% CO_2_. Grown wells were expanded to 96-well culture plates (Nunc, Thermo Fisher Scientific). The culture supernatant of single-clones was evaluated according to the product concentration and best performing clones were further expanded, banked and analyzed.

#### Cell counting and viability

The cell density of CHO-K1 cell lines was calculated by counting the cell nuclei of lysed cells with the particle counter Multisizer 4 (Beckman Coulter). Cell viability was determined by trypan blue exclusion method using a Neubauer cell counting chamber. Growth rate μ [1/d] was calculated according to Equation (1) where X [cells] represents the total viable cell number and t [d] the time in days.

(1) \begin{equation*} \mu = ln(X_1 /X_0 )*1/(t_1 - t_0 ) \end{equation*}

Specific productivity qP [pg/cell/day] was calculated according to Equation (2) where P [pg] represents the product amount and X [cells] the total viable cell number.

(2) \begin{equation*} qP = ((P_1 - P_0 )/(X_1 - X_0 ))*\mu \end{equation*}

#### Clone stability

The clone stability of the CHO-K1/CN54gp140 and CHO-K1/PG9 recombinant cell lines was monitored in shake flasks (Corning) over a period of 20 passages (71–73 days) after thawing in ActiCHO SM medium (GE Healthcare Life Sciences) without selection pressure. Cells were passaged into fresh medium every 3–4 days to 2×10^5^ cells/ml. The cell concentration, viability as well as product concentration was monitored before each splitting.

#### Fed-batch

Fed-batch experiments were performed using the ActiCHO media system including feed A and B (GE Healthcare Life Sciences). Fed-batches were carried out in shake flasks (Corning) with a starting volume of 45 ml and in the DASGIP Bioblock Bioreactor System (4 × 1.2L, DASGIP, Eppendorf AG) with a starting volume of 650 ml and a starting cell concentration of 2–3×10^5^ cells/ml. ActiCHO P medium supplemented with 8 mM L-glutamine served as batch medium, while feed A and feed B were fed to the culture daily starting on day 4. Feed B feed rate was calculated according to the current volume of the culture and was 0.28% (vol/vol) per day. Feed A feed rate was calculated according to the D-glucose concentration of feed solution A and the current D-glucose concentration of the culture and set daily to 6.5 g/l using feed A. The cell concentration, viability, product concentration as well as the glucose concentration was analyzed daily.

### Product concentration

The product concentrations of culture supernatants were determined using enzyme linked immuno-sorbent assays (ELISA).

#### Anti-human IgG-Fc ELISA

96-well immuno-sorbent plates (Nunc MaxiSorp, Thermo Fisher Scientific) were coated with 100 μl of goat anti-human IgG-Fc (Sigma-Aldrich) diluted to 1 μg/ml in 1x Dulbecco's phosphate-buffered saline (DPBS) (Gibco, Life Technologies) and were incubated overnight at 4°C without shaking. Next day wells were blocked with 5% bovine serum albumin (BSA) (Sigma-Aldrich) in 1x DPBS after a washing step. 1x DPBS containing 0.05% Tween 20 (Sigma-Aldrich) was used as washing buffer. After a further washing step, standard (Fc from human plasma, Calbiochem) and samples were added to the blocked microwells and incubated for one hour. As detection antibody horseradish peroxidase conjugated protein A (ProtA-HRP) (Sigma-Aldrich) diluted 1:80 000 in 2% BSA in 1x DPBS was used. As a colorigenic substrate 3,3′,5,5′-tetramethylbenzidine (TMB) was used (Sigma-Aldrich). The reaction was stopped after 20 minutes by addition of 30% H_2_SO_4_ and absorbance was measured at 450 nm (620 nm reference wavelength) in a microplate reader (TECAN Infinite 200 Pro). A sigmoidal curve from standard was calculated and plotted with GraphPad Prism5.

#### Anti-human IgG-λ ELISA

We used the same protocol as described above for IgG-Fc, except that we used goat anti-human lambda HRP conjugate (Southern Biotech) as a detection antibody in a 1:2000 dilution. IgG1 lambda from human myeloma plasma (Sigma-Aldrich) was used as a standard.

#### CN54gp140 ELISA

96-well immuno-sorbent plates (Nunc MaxiSorp, Thermo Fisher Scientific) were coated with 100 μl Lectin from *Galanthus nivalis* (Sigma-Aldrich) diluted to 5 μg/ml in coating buffer at 4°C overnight. 50 μl of samples and standards, diluted in dilution buffer in a 1:2 dilution series, were transferred to the pre-coated plate and incubated for one hour on a shaker at 300 rpm. A_280_ quantified affinity purified CN54gp140 was used as standard at a starting concentration of 100 ng/ml. Next, the plate was incubated with 50 μl of monoclonal anti-gp41 (HIV-1) IgG1 5F3 (Polymun Scientific, Austria) diluted to 1 μg/ml in dilution buffer for one hour. Afterward, the plate was incubated with 100 μl of HRP conjugated, polyclonal, γ-chain specific goat anti-human antibody. The color reaction was induced by adding 100 μl of staining buffer (1.5 mg/ml o-phenylene diamine diluted in 0.15 M citric acid buffer, pH 5.0 containing 0.01% H_2_O_2_) to each well. After incubation for 5 min, the reaction was stopped by the addition of 100 μl 25% H_2_SO_4_ to each well. Absorption was measured at 492 nm (620 nm reference wavelength) using a microplate reader, and mean concentrations were calculated relative to the standard curve.

#### PG9 binding assay

To test the binding specificity of the produced PG9 antibody, 96-well immuno-sorbent plates (Nunc MaxiSorp, Thermo Fisher Scientific) were coated with 100 μl gp120 ZM109 (kindly provided by Dr. Lukas Mach, University of Natural Resources and Life Sciences, Vienna) diluted to 1 μg/ml in coating buffer overnight at 4°C. After each incubation step the plates were washed three times using washing buffer. PG9 antibody as well as negative control 2F5 (Polymun Scientific, Austria), a monoclonal anti-gp41 HIV-1 antibody, were diluted to a starting concentration of 1 μg/μl in dilution buffer and further diluted in a 1:2 dilution series. 50 μl of the dilutions were transferred to the pre-coated plates and incubated for one hour on a shaker at 300 rpm. Afterward, the plates were incubated with 100 μl of HRP conjugated, polyclonal, γ-chain specific goat anti-human antibody (Life Technologies) and stained as above described for CN54gp140 ELISA.

### Real-time PCR analysis

#### Sample preparation for analysis

Genomic DNA and RNA were isolated from stable transfected cells. RNA was isolated with TRIzol Reagent (Life Technologies) according to the manufacturer's instructions. RNA integrity was assessed by loading each sample to a MOPS gel. RNA was treated with DNaseI (Thermo Fisher Scientific) prior to reverse transcription by RevertAid First Strand cDNA Synthesis Kit (Thermo Fisher Scientific). Genomic DNA was isolated by QIAamp DNA Blood Mini kit (Qiagen) according to the manufacturer's instructions.

IgG-Fc DNA and cDNA were detected by a TaqMan assay. As a genomic control *Actb* was used. TaqMan probes as well as qPCR Core kit were ordered from Eurogentec. PG9 antibody heavy and light chain DNA and cDNA were detected by a Sybr Green assay. We used Taq polymerase from 5PRIME for this assay. Primers were ordered from Eurofins Genomics. As a template 5 ng genomic DNA or cDNA derived from 50 ng total RNA was used per reaction in a 25 μl final volume. See probe and primer sequences under the headline ‘Primer and probe list’.

#### Calculation of integrated gene copy numbers and mRNA expression levels

Integrated gene copy numbers (gcn) as well as relative transcript levels were calculated with the 2^−ΔΔ*C*^_T_ method (29). IgG-Fc/ PG9 HC/ PG9 LC integrated copy numbers and mRNA expression (cDNA) levels were measured by real-time PCR using the genomic *Actb* and *Actb* cDNA as a reference for relative quantification. All gcn and relative transcript values in this study are indicated in x-fold values relative to genomic *Actb* and *Actb* transcript. PCR amplification efficiency ranged from 96% to 100%.

### Sodium dodecyl sulphate-polyacrylamide gel electrophoresis (SDS-PAGE)

SDS-PAGEs were performed using the XCell Sure Lock Minicell electrophoresis system and precast gels (Life Technologies). The increase of product during fed-batches was determined on NuPAGE 4–12% bis-tris gels by applying 15 μl of reduced (10% (vol/vol) 0.5 dithiothreitol (DTT)) or non-reduced culture supernatants as well as specific standards. Gels were stained using SYPRO Ruby protein gel stain (Life Technologies) and fluorescence was detected using a biomolecular imager (ImageQuantTM LAS4000, GE Healthcare Life Sciences). The quality of CN54gp140 was determined on a NuPAGE 3–8% tris-acetate gel by applying 20 μg of non-reduced, purified product. The gel was stained using Coomassie brilliant blue.

### Primer and probe list

Real-time PCR analysis

TaqMan probes:

TaqMan IgG-Fc: 5′ 6-FAM – ACAAGTGCAAGGTGAGCAACAAGGC - BHQ-1 3′

TaqMan ACTB: 5′ 6-FAM – CCATCCTGGCCTCACTGTCCACCT – TAMRA 3′

Primers:

IgG-Fc for: CACCAGGATTGGCTGAATG

IgG-Fc rev: TGGAGATGGTCTTCTCGATG

ACTB for TaqMan: TGAGCGCAAGTACTCTGTG

ACTB rev TaqMan: TTGCTGATCCACATCTCCTG

PG9 HC for Sybr: ACCGTCAGTCTTCCTCTTCC

PG9 HC rev Sybr: TCCTTGCCATTCAGCCAGTC

PG9 LC for Sybr: CTGCAATGGAACCAGCAATG

PG9 LC rev Sybr: TCTCGTGCTTGTCAGAGACT

ACTB for Sybr: CGTACCACTGGCATTGTGAT

ACTB rev Sybr: GGCAACATAGCACAGCTTCT

The following primers have been used to amplify the homology regions for BAC recombination by ET-cloning:

Rosa 3 HR exon2 for: TTTTTTCTCGAGGTCTTGTGACACTCAAGAAGCTACC

Rosa 3 HR exon2 rev: TTTTTTTTAATTAAGCTGATTCTGCAGGCCGACTTCAG

Rosa 5 HR exon2 for: TTTTTTGCGATCGCCTGCATGCAGTGGAAACACTCTTGTC

Rosa 5 HR exon2 rev: TTTTTTCCTTAGGGGAGATGTTTCTAAAAGGACCAACAGTC

Rosa 3 HR exon9 for: TTTTTTCTCGAGCAGATATGCCATTTGGAAAAAGGT

Rosa 3 HR exon9 rev: TTTTTTTTAATTAACTGTCCAAGAAGCAAGTGCTTCTC

Rosa 5 HR exon9 for: TTTTTTGCGATCGCCTTGGGAAAATATATTGAAATATC

Rosa 5 HR exon9 rev: TTTTTTCCTTAGGATCCACAGAAGCAGTTCTCAGTG

Rosa 3 HR Thumpd for: TTTTTTCTCGAGGGAGTGCCGTGCGCCGCAGCCCG

Rosa 3 HR Thumpd rev: TTTTTTTTAATTAACTTCTTTCCCCCGGGGCCCGGTCGTG

Rosa 5 HR Thumpd for: TTTTTTGCGATCGCCGCTCAGAGACTCACGCAGCCCT

Rosa 5 HR Thumpd rev: TTTTTTCCTTAGGGCGAGACTCGAGTTAGGCCCAACG

Hprt 3 HR for: TTTTTTCTCGAGGGCTAGATGCCCCATGAGGGCGGCG

Hprt 3 HR rev: TTTTTTTTAATTAACTGATCCTTCCTGAAGCCGCCCTCCG

Hprt 5 HR for: TTTTTTGCGATCGCGCCCAGCGGAGCCTCCGGGGACGGAGCC

Hprt 5 HR rev: TTTTTTCCTTAGGGGACCGGTCGGCTCGCGGCAAAAAGCGG

Actb 3 HR for: TTTTTTCTCGAGGATATCGCTGCGCTGGTCGTCGACAAC

Actb 3 HR rev: TTTTTTTTAATTAATGGAAGGGAACAGCCTTCTTAGCAC

Actb 5 HR for: TTTTTTGCGATCGCCTGCCCTAGGTCCGCCTCCGGGCC

Actb 5 HR rev: TTTTTTATTTAAATGGCGAACTATCAAGACACAAAAGAAG

Rps21 3 HR for: TTTTTTCTCGAGACGACGCCGGCGAGTTTGTGGACC

Rps21 3 HR rev: TTTTTTTTAATTAATCATCTGGATGGACGCGTGGTCCTTG

Rps21 HR for: TTTTTTGCGATCGCGCTGGACAAGTGAGCAGGTAGGCC

Rps21 HR rev: TTTTTTATTTAAATCCTGAGGCCGTCCCTGCAGAGTTAC

### Sequence information for genetic elements

Rps21 5′ UTR (*Mus Musculus*):

CCCTCCTGCCCAATAGTGCTGCGGAGGCACGAGCTACTTCCTTTCTGCGCT CTCGCTGGACAAGTGAGCAGGTAGGCCTCGGTGCGGGCTGTGTGCGAGCGGGGC CGGGCTGGGCCGGGCTGGGCGCAGTTGGGGAGGTCGCGGTCACATGGCCGAGAG TGCTTGCGGGCCATGGGTCTGGTTGGGCCCGCAGACTCGGAGTGCGGGCCTGAC CAGTCTCAAGACCTGCGTGGTAACTCTGCAGGGACGGCCTCAGGATG

Artificial intron (Clontech):

GAATTAATTCGCTGTCTGCGAGGGCCAGCTGTTGGGGTGAGTACTCCCTCT CAAAAGCGGGCATGACTTCTGCGCTAAGATTGTCAGTTTCCAAAAACGAGGAGG ATTTGATATTCACCTGGCCCGCGGTGATGCCTTTGAGGGTGGCCGCGTCCATCT GGTCAGAAAAGACAATCTTTTTGTTGTCAAGCTTGAGGTGTGGCAGGCTTGAGA TCTGGCCATACACTTGAGTGACAATGACATCCACTTTGCCTTTCTCTCCACAGG TGTCCACTCCCAGGTCCAACTGCAGGTCG

## RESULTS

### Optimization of BAC-based vector design

We used two strategies to design BAC-based expression vectors appropriate for recombinant protein production. First, we searched for BACs containing a permissive chromatin environment and combined them with strong ectopic promoters. Second, we searched for BACs containing loci highly transcribed in mammalian cells and expressed the protein of interest under the endogenous promoter. For the first approach, we decided to use two BACs harboring the murine *Rosa26* and *Hprt* (hypoxanthine phosphoribosyltransferase) loci. The *Rosa26 and Hprt* loci are well known to contain permissive chromatin and are widely used in the mouse transgenic field for targeted transgene integration (30–33). We constructed a series of plasmids combining different conventional promoters (Caggs, Ef1α, Ubiquitin C, CMV and SV40) expressing the Fc part of human IgG1 (IgG-Fc) as a model protein and recombined them into the *Rosa26* and *Hprt* BACs (Figure 1a and b). Regarding the second approach, we used BACs harboring the murine *Actb* (β-actin) and *Rps21* (ribosomal protein 21) loci, which are highly transcribed in mammalian cells. The incoming (promoterless) constructs were inserted in frame to the translational start site of the *Actb* and *Rps21* genes by recombineering (27,28), thus the IgG-Fc protein was expressed under control of the respective endogenous promoters (Figure 1a).

To test the performance of the different BAC-based expression vectors we transfected them into CHO-DUKX-B11 (34) suspension cells. Plasmids with identical expression units were used as controls. We selected transfected cells for three weeks with neomycin and analyzed secreted protein production levels in the stable cell pools. The use of the *Rosa26* BAC backbone (BAC^Rosa26^) in combination with Caggs promoter resulted in the highest protein expression levels in the cell pools (22.7 ± 1.2 pg/cell/day [pcd], values represent mean ± SEM) followed by the constructs combining the BAC^Rosa26^ with the Ef1α promoter (13.9 ± 2.8 pcd) and the *Hprt* BAC backbone (BAC^Hprt^) combined with the Caggs promoter (12.7 ± 6.4 pcd, Figure 1c). Of note, cell pools generated with the respective control plasmids showed considerably lower productivity. Cell pools generated with combinations of the BAC^Rosa26^ and the BAC^Hprt^ with the CMV, the Ubiquitin C and the SV40 promoters, as well as the *Actb* and *Rps21* constructs yielded only moderated expression levels (Figure 1c). Next, we performed a direct comparison of the strength of each construct by normalizing the secreted protein and transcript levels to the integrated vector copy number in the cell pools. The combination of the BAC^Rosa26^ and the Caggs promoter proved to be the best choice regarding normalized transcription compared to all the other constructs, but mostly we did not find striking differences between the *Rosa26* and the *Hprt* BACs (Figure 1d). Interestingly, we observed an overall lower copy number integration for the BAC^Hprt^ constructs (1.9 relative copies on average) compared to the BAC^Rosa26^ constructs (5.2 relative copies on average) in the stable cell pools (Figure 1e). This phenomenon could explain why in general the productivity of the cell pools is higher with the BAC^Rosa26^ compared to the BAC^Hprt^ constructs despite of their rather similar normalized protein productivity and transcript levels.

Having identified the BAC^Rosa26^ in combination with the Caggs promoter (Caggs:BAC^Rosa26^) as the best genetic tool, we tested if other genetic elements may improve its performance. We replaced the bovine growth hormone (bGH) polyA by a Simian virus 40 (SV40) polyA, added an artificial intron sequence derived from Clontech and a 5′ untranslated region (UTR) sequence derived from the murine *Rps21* gene to the original construct. The use of these elements did not result in any beneficial effect. Interestingly, the use of an intron sequence downstream of the transgene decreased the expression levels alone and in combination with the 5′ UTR (Figure 1f).

Next, we investigated the clonal distribution within the cell pools generated with the Caggs:BAC^Rosa26^, the Caggs:BAC^Hprt^ and Caggs:plasmid control. We isolated single cell clones by limiting dilution. 250 single-cells were plated of which 45–55 clones (18–22%) were growing and were pre-screened for IgG-Fc production. The best producing 20 clones from each pool were further characterized (Figure 2a). The Caggs:BAC^Rosa26^ derived clones had a productivity ranging from 15 to 87 pcd, while Caggs:BAC^Hprt^ derived clones ranged from 13 to 26.5 pcd. Productivity of the plasmid derived clones was considerably lower (1.8 to 10 pcd). Thus, the BAC-based expression vectors represent a considerable improvement compared to plasmid-based expression vectors. Indeed, stable cell pools generated by electroporation of the Caggs:BAC^Rosa26^ construct expressing eGFP showed that all clones of the pool expressed the transgene (Figure 2b). In addition, the mean fluorescence intensity of the Caggs:BAC^Rosa26^ derived cells was considerably higher compared to the Caggs:plasmid control. We observed two distinct cell populations within the Caggs:plasmid pool. This could reflect an oligoclonal population after cell transfection or that the eGFP negative cells could lose the eGFP expression via transgene silencing which is often observed in plasmid based transgenesis. Furthermore, unlike in the case of plasmids (35,36), copy number analysis of Caggs:BAC^Rosa26^ clones showed a direct correlation between the specific productivity and the integrated vector copy number, ranging from 0.5 to 25.5 copies relative to genomic *Actb* (Figure 2c).

### Fed-batch production of CN54gp140 using an optimized BAC-based vector

To gain further insight into the usefulness of the Caggs:BAC^Rosa26^ vector applied to industrial processes, we produced the HIV-1 (CN54(37)) envelop glycoprotein CN54gp140 in CHO-K1 cells using fed-batch cultures. Of note, the CN54gp140 protein is highly glycosylated (38) and is considered to be difficult to express (39,40). Fermentation of a cell line generated with the Caggs:BAC^Rosa26^ vector using a bioreactor or shake flask yielded up to 1 g/l (Figure 3a and b). For comparison, previous cell lines expressing gp140 (GS amplified) yielded maximum in the single digit mg/l range (39,40). The Caggs:BAC^Rosa26^ CN54gp140 cell line (CHO-K1/CN54gp140) was tested for stability in terms of specific and volumetric productivity by routine cultivation in ActiCHO SM medium without selection pressure for 20 passages (71 days) (Figure 3c). The specific and volumetric productivity stayed constant over the whole period of time with a mean ± SEM of 2.56 ± 0.16 pcd and 13.15 ± 1.24 mg/l. The growth rate was also constant over time and averaged 0.89 ± 0.02 per day. Furthermore, the quality of the affinity purified CN54gp140 was assessed on a non-reducing SDS-PAGE (Figure 3d). Most of the purified product was present in trimers, but also mono-, di- and higher oligomers were detected. These results highlight the stability and high yield of BAC-vectors derived cell lines and provides a proof of concept for its use at industrial level.

### Production of an anti-HIV-1 antibody as a complex protein model using BAC-based vectors

To optimize the BAC-based expression system for production of complex proteins, we chose an antibody as a model protein. PG9 (IgG1-λ) is a neutralizing antibody which targets the variable regions 1 and 2 (V1/V2) of the HIV-1 envelope glycoprotein 120 (gp120) (41). We explored three different strategies to produce an antibody using the BAC expression system. First, we generated two independent expression cassettes for the PG9 light chain (LC) and heavy chain (HC) and placed them at different positions into the BAC^Rosa26^. We tested three different insertion sites to minimize transgene silencing due to transcriptional interference between the expression cassettes. In the BAC 10, BAC 20 and BAC 30 vectors, heavy chain (HC) and light chain (LC) constructs were recombined 10, 20 and 30 kb apart from each other (Figure 4a). Second, we linked the expression of PG9 LC and HC using either the FMDV 2A peptide or an Internal Ribosome Entry Site (IRES) and inserted the resulting expression cassette into a single location into the BAC^Rosa26^ (Figure 4a). Third, PG9 LC and HC expression cassettes were inserted into two different BAC^Rosa26^ vectors and cell lines were generated by co-transfecting both BACs (Figure 4a).

We generated stable cell pools by transfecting all the BAC^Rosa26^ constructs and corresponding control plasmids into CHO-DUKX-B11 cells followed by antibiotic selection. Antibody production in the stable cell pools showed higher yields using BAC vectors compared to plasmids with relatively similar values between different BAC constructs (Figure 4b). All BAC pools produced between 3.52 and 4.59 pcd, with the exception of the EMCV IRES BAC (1.72 pcd). Analysis of transcript expression levels normalized to the integrated vector copy number revealed that all the BAC-based constructs expressed higher levels of HC and LC than their respective control plasmids. Interestingly, we observed that the LC transcripts were more abundant than the HC in all the BACs containing two distinct expression cassettes (Figure 4c).

We then analyzed the clonal distribution of the cell pools generated with the BAC HC and BAC LC (BAC HC/LC), BAC 10, BAC 20, and control plasmid p HC and p LC constructs (p HC/LC). Out of 300 plated single cells, 51–111 clones (17–37%) were growing. 50 clones per construct were pre-screened for antibody production and the best 30 were used to analyze the specific productivity. While single clones derived from the BAC 10 and BAC 20 pools showed little variability regarding their productivity, clones derived from the co-transfection of HC and LC BAC constructs (BAC HC/LC) showed a heterogeneous distribution and the highest expression levels, with the best clone producing 57 pcd (Figure 4d). Of note, the majority of single clones derived from the pool generated with the plasmid control (co-transfection, p HC/LC) did not express detectable levels of the antibody, reaching a maximum specific productivity of 7.18 pcd.

LC expression levels were consistently higher than the HC in cell pools generated with two distinct expression cassettes (Figure 4c). Since unbalanced HC/LC expression ratio may limit the assembly and secretion of antibodies, we analyzed the HC and LC transcript levels of single cell clones derived from the BAC HC/LC pool and correlated to the antibody productivity. We found a strong correlation of the specific productivity of clones with their HC transcript levels (*R*^2^ = 0.9497; *P* < 0.0001) but not with their LC expression levels (*R*^2^ = 0.4203; *P* = 0.0589) (Figure 4e). This suggests that the HC expression is a limiting factor for the production of the PG9 antibody.

**Figure 5.**
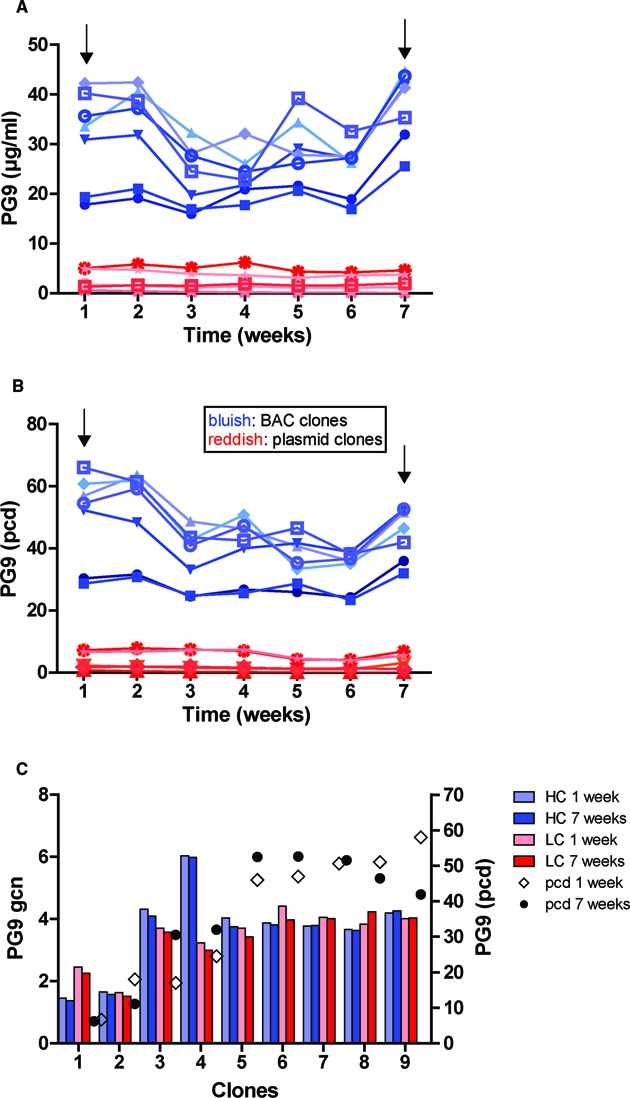
Long-term stability of PG9 antibody producing clones without selection pressure. (**A**) Volumetric (μg/ml) and (**B**) specific productivity (pcd) of BAC (in bluish color) and plasmid (in reddish color) clones generated from BAC HC/LC and p HC/LC stable pools during a 7 weeks culturing period. Arrows indicate sample collection for genomic DNA isolation. (**C**) LC and HC integrated vector copy changes were monitored in 9 BAC^Rosa26^ HC/LC clones. Gene copy numbers relative to genomic *Actb* (gcn) are indicated as bars. Light bars indicate gcn at the first week, dark bars indicate gcn at the seventh week (bluish color, HC; reddish color, LC); specific productivity of each clone is indicated on a dot plot.

We monitored long-term production stability in the absence of antibiotic selection pressure for seven weeks in BAC (BAC HC/LC) and plasmid (p HC/LC) derived clones. Specific and volumetric productivity stayed relative stable during this time period, only a few highly expressing BAC derived clones showed a slight decrease in specific productivity (Figure 5a and b). We further examined the long-term stability of BAC HC/LC clones regarding their integrated vector copy numbers. Transgene copy numbers of HC and LC did not substantially change during this time (Figure 5c).

**Figure 6.**
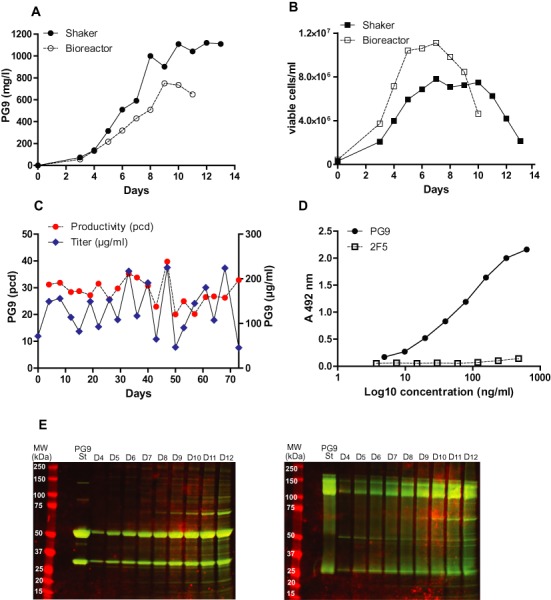
BAC-based PG9 antibody fed-batch production in shake flasks and bioreactors. (**A**) Product accumulation (mg/l) and (**B**) viable cell densities (viable cells/ml) in shake flask and bioreactor fed-batch cultures. (**C**) Specific (pcd) and volumetric (μg/ml) productivity of CHO-K1/PG9 cell line in routine cultivation without selection pressure over 20 passages (73 days). (**D**) Binding curve of PG9 and negative control 2F5 to gp120 (ZM109), PG9 EC50 was calculated to be 64 ng/ml. (**E**) SDS-PAGEs of fed-batch culture supernatants under reducing (left panel) and non reducing (right panel) conditions from CHO-K1/PG9 cell line at different days (D4-D12, PG9 St: antibody standard).

Finally, we assessed the suitability of the BAC-based expression vector system by producing the PG9 antibody using bioreactors and shake flasks. For this purpose, we generated a PG9 producing cell line in CHO-K1 cells using the BAC 20 strategy. This cell line was extensively screened and assessed for its usability in industrial application. PG9 antibody production reached 1.12 g/l in shake flasks and 0.75 g/l in lab-scale bioreactors with 0.8×10^7^ and 1.1×10^7^ maximal viable cell densities in 13 and 11 days fed-batch fermentation processes, respectively (Figure 6a and b). Specific and volumetric productivity of the cell line was stable over 20 passages (73 days) without selection pressure (Figure 6c). SDS-PAGE analysis of supernatants confirmed that PG9 was correctly assembled and secreted. Furthermore, secreted PG9 antibody was functional as shown by a binding assay to its target gp120 (ZM109) (Figure 6e and d). These results offer a proof of principle for industrial production of antibodies using the BAC expression system.

## DISCUSSION

While BAC vectors are commonly used in the mouse transgenic field as expression vectors, their use for recombinant protein production in mammalian cells is still not well established.

Here we describe a comprehensive analysis of BAC-based expression vectors applied to recombinant protein production in CHO cells and report that BAC vectors containing open chromatin regions highly increased recombinant protein production for all proteins tested in this study. However, BAC vectors increase protein production by increasing the transcription of the GOI, thus, they may not offer a significant advantage if non-transcriptional related processes (i.e. translation, folding, transport or secretion) are the limiting factors in the production of the protein of interest.

We found that BAC vectors containing highly transcribed genes such as *Actb* and *Rps21* can be used to express a GOI from the endogenous promoter, albeit resulting in moderated expression levels. On the other hand, the combination of BACs containing permissive chromatin regions with a potent (heterologous) promoter resulted in cell lines expressing high protein levels. Interestingly, transgene expression levels were highly dependent on the combination of open chromatin containing loci and the heterologous promoters. For example, as shown in Figure 1c and d, the SV40 and Ubiquin C promoters (which are well-known to be ‘potent’ promoters) gave poor results when combined with the *Rosa26* or *Hprt* BACs. This illustrates the complexity of chromatin regulation and underscores that even loci which are considered to be permissive or ‘open chromatin’ may not be compatible with some ectopic promoters. Similar observations were obtained by an RMCE study in which the transgene expression levels were dependent not only on the targeting constructs but also on their combination with the genomic integration sites and even on the relative orientation of the transgene (42). This highlights the importance of testing different promoter sequences with different chromatin contexts, since promoter strength can vary broadly between different constructs and cell types (43).

We identified BAC^Rosa26^ in combination with the Caggs promoter as the BAC-based expression vector producing the highest protein and transcript levels. The positive effects mediated by the chromatin environment of *Rosa26* gene are not restricted to one particular cell type or to a particular protein. Beneficial effects have been reported in mouse studies (30), in human cells (HEK 293 (44)) and in hamster cell lines (CHO (45)). By using IgG-Fc as a model protein, we could demonstrate that it is possible to obtain CHO cell pools producing 24 pcd in three weeks using the Caggs:BAC^Rosa26^ vector. In addition, single cell clones producing up to 87 pcd could be isolated by analyzing as few as 50 clones. Furthermore, unlike in the case of plasmids (35,36), the productivity of BAC-based clones was directly proportional to their integrated transgene copy numbers in the host cell. This phenomenon indicates that BACs are able to overcome chromatin positional effects upon stable integration into the CHO cell genome. In addition to IgG-Fc, we showed the advantages of the BAC vectors in the production of HIV-1 envelope glycoprotein CN54gp140. We could reach a yield of 1 g/l in fed-batch cultures by using the BAC^Rosa26^ vector. CN54gp140 is a highly glycosylated protein and considered to be difficult to express. Indeed, productivity of cell lines expressing recombinant gp140 are reported to be mainly in the single digit mg/l range (39,40). Moreover, we could show that the BAC^Rosa26^ vector conferred long-term production stability and optimal protein quality.

We have demonstrated that complex proteins such as antibodies can be successfully expressed from a single BAC-based vector containing two separate expression units placed at different locations within the BAC sequence. However, according to our results, co-transfection of two BAC vectors carrying each one of the polypeptide chains seems to be more beneficial. This could be due to promoter interference when both expression units are placed in the same vector (46) although the distance was 30 kb or may reflect the complexity of antibody production. By placing the HC and LC of the PG9 antibody in the same BAC vector, the ratio between HC and LC expression is fixed which may not be optimal for appropriate antibody folding and secretion (47). On the other hand, expressing HC and LC from two independent BACs may result in different HC/LC expression ratios depending on the number of copies integrated of each BAC. Therefore, it could be easier to find clones with the appropriate HC/LC ratio and antibody expression. Indeed, our results suggest that the expression of the HC is the limiting factor in the production of the PG9 antibody and this should be taken into account in future design of BAC vectors for antibody production. Nevertheless, the use of BAC^Rosa26^ to express the PG9 antibody was much superior to a conventional vector allowing us to isolate single cell clones with a productivity of 57 pcd and more than 1 g/l in bioreactor and shake flasks. Furthermore, binding analysis demonstrated the functionality and quality of the produced PG9 antibody. In addition, we have identified two additional insertion sites within the BAC^Rosa26^ vector that can be used not only to produce complex proteins but also to co-express polypeptides to improve the quality of the end product (e.g. glycosylation modifying enzymes), the cell viability (e.g. antiapoptotic factors), the cell metabolism (e.g. mTOR (48)), or the secretion efficiency (e.g. XBP-1 (49)). Loss of productivity during long-term culture is a common phenomenon in recombinant CHO cells generated with techniques based on random integration and it is attributed both to gene copy number loss and epigenetic silencing effects (36). The use of BACs successfully stabilized long-term protein expression in the absence of selection pressure in all of the tested target proteins. This suggests that BAC^Rosa26^ provides a chromatin environment protecting against transgene silencing. On the other hand, CHO cells are known to exhibit genotypic drift during long-term culture which may result in transgene rearrangement and loss of expression (50). However, we did not observe changes in BAC integrated copy numbers during the long-term culture, suggesting that integrated BAC vectors are genetically stable.

Besides all the advantages described above there are certain points to consider when using the BAC-system. Firstly, the BAC size: large genomic sequences present in the BAC serve as permissive environment for the transgene; however, the large size of the BACs is a disadvantage during cell transfection. DNA size affects the transfection efficiency and the uptake of large DNA molecules may be compromised in some cell types. Secondly, possible BAC toxicity: the open chromatin region present in the BAC has to be chosen carefully. BAC vectors derived from genomic libraries may contain several loci and the over-expression of some of these extra genes can be toxic to the cells. Thus, for certain BACs high copy number integration may be not supported by the host cells. In this regard, we observed that the BAC^Rosa26^ constructs integrated with a mean of 5.2 relative copies in stable cell pools (ranging from 2.4 to 8.8) while the BAC^Hprt^ stable pools harbored only 1.9 relative copies on average (ranging from 0.9 to 3.2) regardless of the heterologous promoter used. In addition, the narrow distribution of IgG-Fc productivity in Caggs:BAC^Hprt^ single cell clones compared to the Caggs:BAC^Rosa26^-derived clones suggests as well that there is a limiting factor for production, presumably the number of vector copies integrated in the cell host. Thirdly, heterogeneous expression: while the BAC-system can help to boost overall protein production, it is very important to match the expression technique to the study needs. The use of BAC-based vectors generates a heterogenic stable pool population with cells harboring different integrated copy numbers and consequently different expression levels. Therefore, if the aim is to study the characteristics, activity or functionality of a protein, targeted integration to an active genomic site is the appropriate method to choose, since it can ensure equal levels of expression in different cell clones. This can be done for example by RMCE technology (22) or by using the CRISPR/Cas9 system (51).

The Caggs:BAC^Rosa26^ construct confers a reasonable (approximately 5 pcd/gcn) specific productivity per relative copy. Based on the RMCE technology others have identified genomic hot-spots in CHO cells supporting a productivity of up to 13 pcd per single transgene integration (23). This indicates that there can be more efficient hot-spots in the CHO genome than the ones used in our study. A future development of the BAC expression technology would be to perform a genomic screening, searching for hot-spots allowing high expression levels in CHO cells (or to use previously identified ones), cloning such genomic regions into BAC vectors and use them as BAC-based expression vectors. This future generation of BAC vectors should be useful tools for recombinant protein production.

In summary, BAC-based vectors with an optimal chromatin environment are valuable tools for recombinant protein production in mammalian cells, since they confer position independent, copy number dependent and high and stable expression. Moreover, high producing stable cell pools and single cell clones can be obtained without the need of transgene amplification in three and six weeks, respectively. Therefore, BAC-based expression vectors provide a fast and efficient alternative to conventional cell line-based production.
